# GTAD: Graph and Temporal Neural Network for Multivariate Time Series Anomaly Detection

**DOI:** 10.3390/e24060759

**Published:** 2022-05-27

**Authors:** Siwei Guan, Binjie Zhao, Zhekang Dong, Mingyu Gao, Zhiwei He

**Affiliations:** School of Electronic and Information, Hangzhou Dianzi University, Hangzhou 310018, China; guansw@hdu.edu.cn (S.G.); zhaobj@hdu.edu.cn (B.Z.); englishp@hdu.edu.cn (Z.D.); mackgao@hdu.edu.cn (M.G.)

**Keywords:** anomaly detection, multivariate time series, graph neural network, temporal convolutional network

## Abstract

The rapid development of smart factories, combined with the increasing complexity of production equipment, has resulted in a large number of multivariate time series that can be recorded using sensors during the manufacturing process. The anomalous patterns of industrial production may be hidden by these time series. Previous LSTM-based and machine-learning-based approaches have made fruitful progress in anomaly detection. However, these multivariate time series anomaly detection algorithms do not take into account the correlation and time dependence between the sequences. In this study, we proposed a new algorithm framework, namely, graph attention network and temporal convolutional network for multivariate time series anomaly detection (GTAD), to address this problem. Specifically, we first utilized temporal convolutional networks, including causal convolution and dilated convolution, to capture temporal dependencies, and then used graph neural networks to obtain correlations between sensors. Finally, we conducted sufficient experiments on three public benchmark datasets, and the results showed that the proposed method outperformed the baseline method, achieving detection results with F1 scores higher than 95% on all datasets.

## 1. Introduction

Industrial equipment and service systems, such as servers, cybersecurity and robotic systems, are often tested using multiple time series (telemetry data or sensor data) to keep track of equipment operation and promptly detect system anomalies. Since anomalies in the data imply important information, timely and efficient detection of these messages by operational engineers helps to improve efficiency, reduce costs and increase safety [1]. Anomalies are system behaviour patterns in time steps that do not conform to a well-defined notion of normal behaviour [2]. The purpose of anomaly detection is to provide the opportunity to take action to identify and resolve potential problems before they cause disasters. The difficulty of label acquisition and the extreme imbalance between normal and abnormal categories have resulted in anomaly detection being classified as an unsupervised machine learning task [3,4,5]. Traditionally, domain experts set thresholds for normal events, and the system is considered abnormal if its measurements exceed the expert-defined thresholds. Given the dramatic increase in the number of sensors required to detect systems today and the hidden correlation and temporal information in the data becoming cumbersome, the traditional threshold approach is no longer applicable and automatic anomaly detection methods have become a necessity. 

Currently, anomaly detection has become an active research topic in the field of data mining and is widely used in areas such as healthcare, aerospace and industrial production [6,7,8,9,10,11]. Although numerous time series anomaly detection methods have been developed for univariate time series [1,12,13,14], where the anomalies are detected mainly based on one specific metric, for a complex real-world system, there is an intrinsic correlation between different sensors. A single univariate time series does not represent the overall state of the system well. Formally, a multivariate time series consists of a set of univariate time series, each of which describes an attribute of a complex entity. Multivariate time series have higher spatial and temporal complexity, more noisy data and more severe disturbances. In addition, there is often synergistic variation among individual features. Dividing multivariate time series into several univariate time series will lead to poor performance in anomaly detection [15,16]. This causes difficulties in anomaly detection for multivariate time series.

In the past few years, many classical algorithms have been proposed for automatic anomaly detection in multivariate time series. These include distance-based methods [17,18], clustering-based methods [19,20], similarity-based methods [21,22] and classification-based methods [23,24], which have driven the development of anomaly detection techniques. However, the failure to consider the temporal dependence of time series limits the performance of these techniques. To address this problem, time series prediction models, such as autoregressive moving average (ARMA) [25] and autoregressive integrated moving average (ARIMA) [26], are used to model time-dependent anomaly detection. However, these methods do not consider the correlation between time series and are sensitive to noise, which affects the robustness of the models. We argue that it is beneficial to improve the performance of the model by modeling the time dependence and the correlation between different sequences. 

Recently, deep learning has gained significant attention in computer vision and natural language processing and has also been applied to the task of anomaly detection. Anomaly detection algorithms for deep learning can be broadly classified into two categories: prediction-based and reconstruction-based. Reconstruction-based anomaly detection algorithms, e.g., TAnoGAN [27], EncDec-AD [28], TCN-AE [11] and OmniAnomaly [29], reconstruct the input by learning the data distribution of the normal state of the time series. Reconstruction errors are used for anomaly detection, avoiding the difficulty of time series prediction; however, minor anomalies in the data are not easily detected using this method. In addition, prediction-based models, such as LSTM-NDT [10] and GDN [30], learn historical patterns to predict the future and perform anomaly detection by prediction errors. Lastly, hybrid models, such as NSIBF [31] and MTAD-GAT [32], use prediction and reconstruction errors of all dimensions for detection. This multi-task optimization problem is extremely complicated when there are numerous features in the data. We believe that combining the advantages of both can help improve detection performance without increasing the complexity of the task.

To address the above problem, we proposed a new framework, namely, graph attention network and temporal convolutional network for multivariate time series anomaly detection (GTAD). Specifically, GTAD uses temporal convolutional networks to capture the temporal dependence of the sequences, where causal convolution maintains the causality of the sequences and dilation convolution allows for flexible perceptual field sizes. Subsequently, graph attention networks are adopted to model the correlation of different time series, which are naturally obtained through the properties of the edges in the structure of the graph. Finally, we utilized a joint approach based on the prediction and reconstruction of one feature to optimize the model, simplifying the optimization objective.

We summarize the study’s main contributions as follows:

1. We proposed a new framework for an unsupervised multivariate time series anomaly detection algorithm (GTAD) that combines the advantages of prediction-based approaches, which focus on feature engineering at the next time step, and reconstruction-based approaches, which emphasize capturing the overall distribution of the data.
2. GTAD uses parallel operations instead of RNN frameworks, such as LSTM and GRU, and its ability to extract contextual information is enhanced, resulting in a model with low sensitivity to sliding window size.
3. GTAD specifies the optimization objective by using the error of prediction and reconstruction for one dimension as the loss function, rather than all dimensions, leading to better detection performance.

The structure of the rest of the paper is as follows. The related work on the anomaly detection of time series is described in Section 2. The method overview is introduced and briefly analyzed in Section 3. In Section 4, the effectiveness of the proposed method is experimentally verified. Finally, we summarize the whole paper and suggest possible future work in Section 5.

## 2. Related Works

Multivariate time series anomaly detection algorithms have been advanced by many methods that can achieve effective detection in recent years. Here, we summarize these anomaly detection methods as classical methods and deep learning-based methods.

Classical methods: The K-nearest neighbor (KNN) [17] algorithm calculates the average distance of the K nearest neighbors of each sample as the anomaly score. However, the computational complexity of this method is high when the feature dimension of the data is high. Principal component analysis (PCA) [33] and partial least squares (PLS) [34] are two linear model-based approaches for anomaly detection. However, these models assume that the data are Gaussian-distributed and are only feasible for highly correlated data. The find-CBLOF algorithm [35] assigns a predefined anomaly score known as the cluster-based local outlier factor (CBLOF) for each data instance to cluster different data samples. The extended fuzzy C-means clustering technique [20] can work better to detect anomalies. However, the computational overhead of clustering methods is high, and performance depends heavily on the effectiveness of capturing the clustering structure of normal instances. ARMA [25] and ARIMA [26] are two common statistical techniques that are used for anomaly detection, which rely on assumptions that the data is generated from a particular distribution. This assumption often does not hold, especially for multivariate time series. Other machine learning methods, such as support vector domain description (SVDD) [36], one-class support vector machine (OC-SVM) [37] and isolation forest (IF) [38], show effectiveness regarding anomaly detection, but these methods do not consider the time dependence, resulting in poor detection of contextual anomalies.

Deep-learning-based methods: Most contemporary state-of-the-art technologies employ some form of deep learning. The DAGMM [39] uses a deep autoencoder to generate a low-dimensional representation and reconstruct the error for each input data, further feeding it into a Gaussian mixture model. However, this method cannot exploit temporal information. The TCN-AE [11], combining the temporal convolutional network (TCN) [40] and autoencoder (AE), was designed to learn the compressed expression of normal time series. This approach has the ability to obtain temporal information but ignores the correlation between time series. The MSCRED [41] constructs a multi-scale signature matrix, reconstructs the matrix using an attention-based mechanism Conv-LSTM and a convolutional encoder–decoder, and detects anomalies using the residual signature matrices. Although correlations and temporal information in multivariate time series are captured using this method, it consumes a lot of training time and works poorly in the case of insufficient data.

Models with LSTM or GRU require long training times. The NSIBF [31] designs an LSTM-based neural network framework for system identification and Bayesian filtering for robust anomaly detection by recursively tracking the uncertainty of the hidden states of the system over time. Ergen et al. [42] proposed an algorithm to turn a variable-length data sequence into a fixed-length sequence using LSTM, followed by an anomaly detector decision function based on a single class support vector machine or support vector data description algorithm. The LSTM-NDT [10] method is an LSTM-based neural network model that makes predictions for each input timestamp. This work also proposes a nonparametric dynamic error thresholding strategy that uses the moving average of the prediction error sequence to set the threshold of anomaly markers. The MAD-GAN [43] uses LSTM-RNN as the basic framework of the GAN to capture potential interrelationships between time series and determines whether a sample is anomalous or not using trained discriminator and reconstruction errors. The MTAD-GAT [32] uses a graph attention networks (GATs) [44] in each of the time dimension and feature dimensions to better represent the complex dependencies of the time series, and then captures the dependency information of the input data through a GRU, which is used for prediction and reconstruction. However, these RNN-based models tend to be inefficient in modeling long time series, especially when the data is noisy.

More recent methods, such as USAD [45], GDN [30] and TranAD [46], do not use resource-hungry recurrent models, but only attention-based network architectures [47] to improve training speeds. The USAD [45], which is based on generative adversarial networks and an autoencoder, is used for unsupervised anomaly detection, where reconstruction errors containing anomalous inputs are amplified by an adversarial trained autoencoder. This is one of the first works to focus on a low overhead, allowing a several-fold reduction in training time compared with existing techniques. The GDN [30] combines structural learning with GAT, applying attention mechanisms to adjacent sensors on the graph to learn predictions for each timestamp and detect anomalies using prediction errors. However, existing graph-neural-network-based anomaly detection methods still have difficulties in dealing with lengthy and highly correlated multivariate time series. The deep-transformer-network-based anomaly detection and diagnosis model (TranAD) [26] employs an attention-based sequential encoder to rapidly make inferences using extensive temporal trend information in the data. 

## 3. Method Overview

In this section, we present the problem in Section 3.1. In Section 3.2, the proposed model GTAD is elaborated. Specifically, GTAD uses TCN, GAT and attention mechanisms to predict and reconstruct the inputs and obtains anomaly scores using the prediction and reconstruction errors. The automatic threshold selection strategy is described in detail in Section 3.3.

### 3.1. Problem Statement

**Figure 1:**
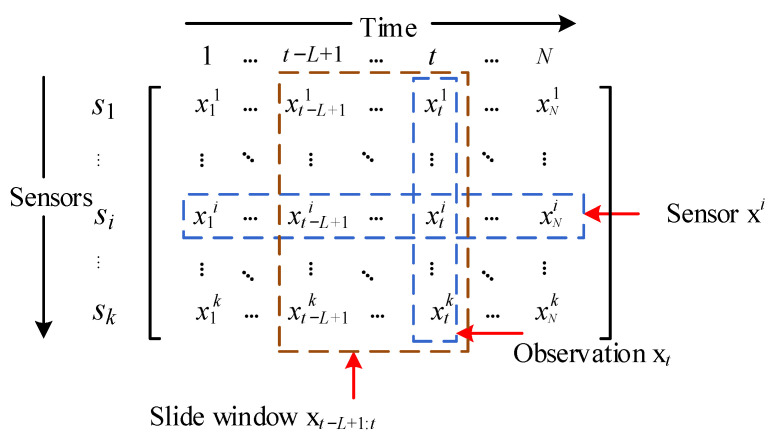
Data formulation of the multivariate time series. Each row x*^i^* represents the measurement data of a sensor and each column *x_t_* represents an observation.

The time series contains observations at successive equal time intervals, and our research objective is a multivariate time series defined as ***X*** ∈ *R^N^*^×*k*^, where *N* denotes the length of the time series and *k* denotes the dimension of the sample at a certain moment, as shown in Figure 1. ***X****_T_* ∈ *R^M^*^×*k*^ is the training set, where *M*(*M* < *N*) denotes the length of the training set and the rest as the testing set, where the training set is full of normal samples and the testing set contains normal and abnormal samples. The input of GTAD is a sliding window of data that is denoted as *X_L_* ∈ *R^k^*^×*L*^, where *L* denotes the length of the sliding window. We define the PR-score as the anomaly score and choose the best threshold using an adaptive threshold strategy. If the PR-score of an instance exceeds the threshold, it is marked as abnormal; otherwise, it is marked as normal.

### 3.2. Model Architecture

**Figure 2:**
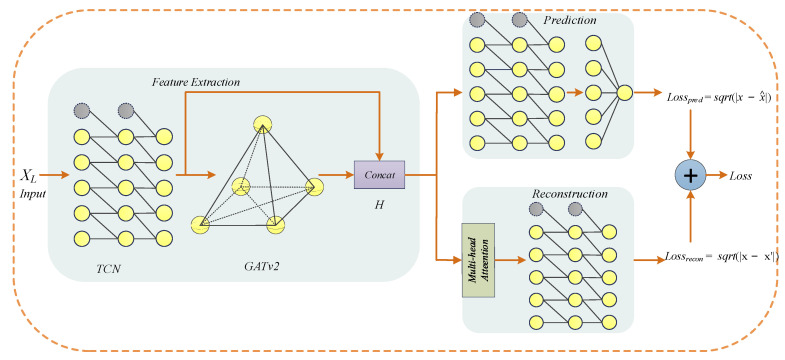
The overall framework of GTAD for multivariate time-series anomaly detection.

GTAD is divided into three parts in total: feature extraction, prediction and reconstruction, as shown in Figure 2. Pseudocode for the training period of the proposed GTAD model is given in Algorithm 1. In the feature extraction, the data are processed in the first step using TCN to obtain the local features and temporal information of the time series, and in the second step by a variant of the graph attention network, namely, GATv2 [48], to process the correlation between different time series. Then, the processed outputs are concatenated to obtain *H* ∈ *R^2k×L^*. In the prediction, *H* is fed to the TCN, followed by a fully connected layer for single-step prediction. In the reconstruction, the complex contextual information of the multivariate time series is processed using a multi-head attention mechanism and is later input to the TCN to obtain the overall reconstruction. The overall loss function is obtained by weighting the prediction and reconstruction errors.
**Algorithm 1:** GTAD Training Algorithm **Input:** Training Dataset X = {x_1_, …, x*_M_*}, The number of epochs R **Output:** Trained GTADGTAD←initialize weightepoch←1**repeat**  **for** *t* = *L* to *M*
**do**    $x\prime_{t}^{i}$$rx_{t-l:t-1}^{\prime i}$← TAD (x_t-L:t-1_)    
$Loss=\sqrt{x_{t}^{i}-x\prime_{t}^{i}}+\sqrt{\overset{t-1}{\underset{j=t-L}{\sum }}\left(x_{j}^{i}-x\prime_{j}^{i}\right)}$
    GTAD←update weight using Loss   
**end for**
  epoch←epoch + 1**until** epoch = R

#### 3.2.1. Temporal Convolution Network

**Figure 3:**
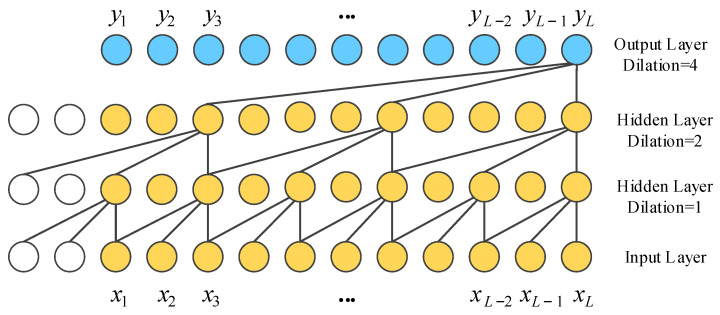
The structure of dilated causal convolution with dilation factors *d* = 1, 2, 4 and kernel size *k* = 3, where *x* denotes the input and *y* denotes the output.

The TCN is a sequential model that combines simplicity, autoregressive prediction and residual connectivity to adapt to long sequence tasks while reducing the computational complexity. The TCN is based on two principles: the network produces an output of the same length as the input and there can be no information leakage from the future into the past. To accomplish the first point, the TCN uses a 1D fully convolutional network framework and zero padding to make the sequence length constant in each layer. To achieve the second point, causal convolution is used to ensure that the output at time *t* is only associated with the input at the current and previous times. To view valid historical information from the distant past, the TCN employs dilation convolution so that the perceptual field is amplified for long sequence tasks rather than recalling temporal information at a linear size over the network depth. The dilation causal convolution is shown in Figure 3.

**Figure 4:**
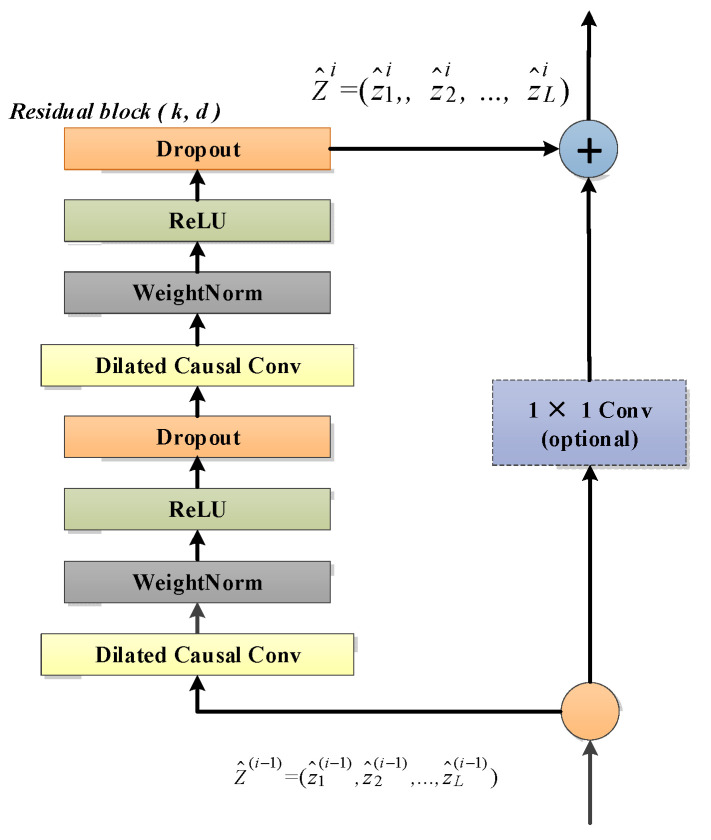
A residual block has two layers of dilated causal convolution, weight normalization, ReLU activation function and spatial dropout, as well as residual connectivity.

Weight normalization, ReLU activation function and a spatial dropout are added after the dilated causal convolution. Finally, unlike the standard ResNet [49] where the input is directly added to the output of the residual block, the TCN uses a 1 × 1 convolution as a residual connection to ensure that the input and output have the same shape, and the residual connection is employed to avoid gradient vanishing in very deep networks. The residual block of the temporal convolutional network is shown in Figure 4. Compared with GRU and LSTM, the TCN can achieve parallel operation without sequential processing, like an RNN framework, and stable gradients can be obtained. In multivariate time series anomaly detection, temporal information can be obtained naturally by the network. Specifically, local mutations that may contain anomalous patterns are very sensitive in convolutional operations. In addition, flexible perceptual field sizes are important for handling sequences containing complex and lengthy temporal patterns.

#### 3.2.2. Graph Attention Network

A GAT is one of the most popular graph neural networks and is considered to be the most advanced graph representation learning architecture. The graph represents the relationships between entities in the network. A graph is formulated as *G* = (*V*, *E*), where *V* is the set of nodes and *E* is the set of edges. We use *k* to denote the number of nodes in a graph. Let *v* ∈ *V* denote a node and *e* = (*v*, *u*) ∈ *E* denote an edge pointing from *u* to *v*. The neighborhood of a node *v* is defined as *N*(*v*) = {*u* ∈ *V*| (*v*, *u*) ∈ *E*}. The adjacency matrix to represent this directed graph, denoted as *A*∈*R^k×k^* with *A_ij_* = c > 0 if (*v_i_*, *v_j_*) ∈*E* and *A_ij_* = 0 if (*v_i_*, *v_j_*) ∉ *E*. Since without a priori knowledge, we do not know the mathematical expression of the adjacency matrix, it will be learned by our model.

The data between different sensors in a system are not isolated, but there are dependencies. The linear and nonlinear dependencies between different sensors can be successfully modeled as graph-structured data. We used a variant of the graph attention network, namely, GATv2, to learn these complex dependencies. The input of GATv2 is the set of node feature vectors, denoted as *V* = {*v*_1_, *v*_2_,..., *v_k_*}, where *v_i_* ∈ *R^F^* and *F* denotes the dimensionality of each node vector. The GATv2 outputs a new set of node feature vectors, namely, *V’* = {*v_1_’*, *v_2_’*,..., *v_k_’*}, where *v_i_’* ∈ *R^F^^’^*. In order to obtain sufficient expressiveness, we transformed the input feature vectors into higher-level feature vectors using a learnable linear transformation, and the output of each node can be expressed as Equation (1).

(1) $$v_{i}{}^{\prime }=sigmoid\left(\underset{v_{j}\in N\left(v_{i}\right)}{\sum }\alpha_{ij}Wv_{i}\right)$$

where *v_i_’* denotes the output of node *v_i_*; sigmoid denotes the sigmoid activation function, *N* (*v_i_*) denotes the set of nodes adjacent to node *v_i_*; α*_ij_* denotes the attention score, which indicates the importance of neighboring nodes to node *v_i_*; and *W*∈*R^F’^*^×*F*^ is the learnable parameter.

The attention score α*_ij_* can be calculated using the following equation.

(2) $$e\left(v_{i},v_{j}\right)=a^{T}LeakyReLU(W[v_{i}||v_{j}])\;\alpha_{ij}=soft\max_{j}(e\left(v_{i},v_{j}\right))=\frac{exp(e\left(v_{i},v_{j}\right))}{\sum_{j\prime \in N_{i}}exp(e\left(v_{i},v_{j\prime }\right))}$$

where *T* denotes transposition, ‖ is the concatenation operation, *a* ∈ *R^2F^^’^* is a learnable parameter and *LeakyReLU* is an activation function. The attention scores α*_ij_* are obtained by normalizing the attention coefficients *e* (*v_i_*, *v_j_*) of all its neighbors *v_j_* ∈ *N* (*v_i_*) using the softmax function.

**Figure 5:**
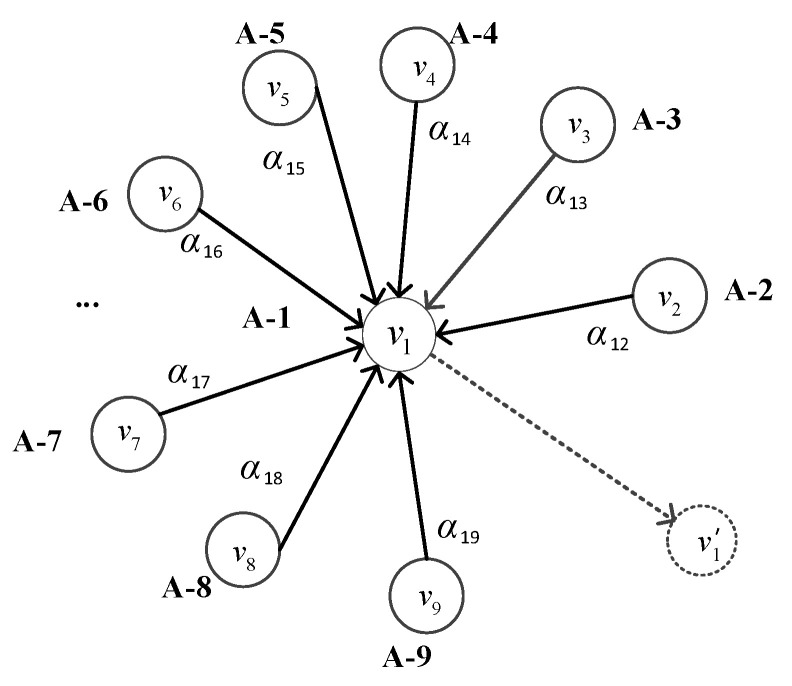
Graph structure of the multivariate time series, where A-1 to A-9 denote the names of univariate time series, *v*_1_ to *v*_9_ denote their corresponding vectors, respectively, and *v*_1_’ is the output vector after the *v*_1_ update.

In the task of multivariate time series anomaly detection, we viewed the whole sliding window *X_L_* as a graph, as shown in Figure 5. Each node represents a sensor datum and the relationship between two sensors is represented by an edge. The information delivery between nodes is achieved through the properties of edges. The update of each node will aggregate the information of the nodes adjacent to it. The interrelationships between time series are learned by the GATv2.

#### 3.2.3. Loss Function 

The prediction-based and reconstruction-based methods were optimized to be adopted by GTAD in Figure 2. In the prediction, we used the autoregressive prediction property of TCNs to perform a single-step prediction for each sliding window. Since a TCN is only suitable for modeling univariate time series and cannot take into account spatial relationships, we finally used a multi-layer perceptron to solve it. In the reconstruction, on the one hand, to not lose temporal information, we added position encoding, which is beneficial to obtain long-range contextual information later, and on the other hand, to obtain the overall data distribution of each sliding window, we applied a self-attention mechanism and TCNs. The loss function is the sum of the prediction and reconstruction errors in one feature dimension, as shown in Equation (3).

(3) $$Loss=\sqrt{x_{t}^{i}-x\prime_{t}^{i}}+\sqrt{\sum_{j=t-L}^{t-1}\left(x_{j}^{i}-x\prime_{j}^{i}\right)}$$

where the first term of the loss function represents the root-mean-square error (RMSE) of the prediction loss in one feature dimension, and the second term represents the RMSE of the reconstruction error in the same dimension. In the absence of prior knowledge of the task, the first feature dimension is chosen by GTAD.

### 3.3. Automatic Threshold Selection Strategy

The PR-score measures the prediction and reconstruction error at each timestamp by Equation (4) as the anomaly score, denoted as ***e*** = {*e*_1_, *e*_2_, …, *e_m_*}. Unlike the loss function, the anomaly score is derived from the two adjacent periods of GTAD.

(4) $$e_{t}=\sqrt{x_{t}^{i}-x\prime_{t}^{i}}+\theta \sqrt{\sum_{j=t-L+1}^{t}\left(x_{j}^{i}-x\prime_{j}^{i}\right)}$$

where the first term is the prediction-based anomaly score, the second term is the reconstruction-based anomaly score and *θ* is the hyperparameter that mediates the weights of the two anomaly scores. In Section 4.6, we present the analysis results of the effect of different values of theta on the performance of the model.

GTAD uses a nonparametric dynamic error thresholding (NDT) strategy [10] to set thresholds and select the best threshold to identify extreme values without labels and without making any assumptions. This approach adapts to data streams with different properties and different ranges, addressing diversity, non-stationarity and noise through automatic thresholding schemes. The threshold is chosen from the set ***ε***, which is expressed as Equation (5). The threshold value *ε* is determined using Equation (6).

(5) ε=μ(e)+zσ(e)

$$\varepsilon =argmax\left(\varepsilon \right)=\frac{\frac{\Delta \mu \left(e\right)}{\mu \left(e\right)}+\frac{\Delta \sigma \left(e\right)}{\sigma \left(e\right)}}{\left|e_{a}\right|+{\left|E_{seq}\right|}^{2}}$$

such that

(6) $$\Delta \mu \left(e\right)=\mu \left(e\right)-\mu \left(\left\{e\in e|e<\varepsilon \right\}\right)\;\Delta \sigma \left(e\right)=\sigma \left(e\right)-\sigma \left(\left\{e\in e|e<\varepsilon \right\}\right)\;e_{a}=\{e\in e|e>\varepsilon )\;E_{seq}=continous\;sequences\;of\;e_{a}\in e_{a}\;$$

The evaluated value for *ε* is determined by *z* ∈ **z**, where **z** is the set of positive values representing the number of standard deviations above *μ*(***e***). The value of z depends on the context, but from empirical facts, it works well when z is between 3 and 13. A value of z less than 3 led to an excessive number of false positives in our experiments. This function also penalizes larger values of ***e_a_*** and sequences ***E_seq_*** in the anomaly score to avoid overly greedy behavior that results in a large number of true anomalies going undetected. Pseudocode for the proposed GTAD model in the anomaly detection phase is presented in Algorithm 2.
**Algorithm 2:** GTAD Detection Algorithm**Input:** Dataset X = {x_1_, …, x*_N_*}, parameter θ**Output:** Labels y: {y*_M_*_+1_, …, y*_N_*} **for** t = *L* to *M* **do**  $x\prime_{t}^{i}$ _← *GTAD* (x*_t_*_-*L*:*t*-1_)  **_**$x_{t-l:t-1}^{’i}$← *GTAD* (x*_t-L+1:t_*)  
$e_{t}=\sqrt{x_{t}^{i}-x\prime_{t}^{i}}+\theta \sqrt{\overset{t}{\underset{j=t-L+1}{\sum }}\left(x_{j}^{i}-x\prime_{j}^{i}\right)}$
 **end for** Threshold *λ* = threshold function (*e*_1_, …, *e_M_*) **for** *t* = *M* + 1 to *N* **do**  $xr_{t}^{i}$, _← *GTAD* (x_t-L:t-1_)  **_**$x_{t-L+1:t}^{’i}$← *GTAD* (x*_t-L+1:t_*)  
$e_{t}=\sqrt{x_{t}^{i}-x\prime_{t}^{i}}+\theta \sqrt{\overset{t}{\underset{j=t-L+1}{\sum }}\left(x_{j}^{i}-x\prime_{j}^{i}\right)}$
  **If**
*e_t_* > λ then   **y***_t_* = 1  
**else**
   **y***_t_* = 0   
**end if**
**end for**

## 4. Experimental Evaluation

In this section, we describe the experimental datasets, baseline methods and evaluation metrics. Then, we present the results of the many experiments conducted to show the effectiveness of GTAD in unsupervised anomaly detection.

### 4.1. Datasets

We employed three publicly available datasets in our experiment. The Soil Moisture Active Passive (SMAP) satellite and the Mars Science Laboratory (MSL) rover datasets are two real-world public datasets collected by NASA [46]. The Server Machine Dataset (SMD) is a five-week dataset collected and made publicly available by a large Internet company [29]. It contains data from 28 server machines. SMD is divided into two subsets of equal size: the first half of the data from each machine is the training set and the second half is the test set. Detailed information about the datasets is shown in Table 1. 

### 4.2. Experimental Setup

In the experiments, the machine learning library Scikit-learn, the deep learning framework Pytorch-1.7.1 and Python 3.6 were adopted. The operating system used to implement the experiment was Ubuntu 16.04, the computer configuration was an Intel(R) Core (TM) i7-6850K CPU @ 3.60 GHz and the GPU was an NVIDIA GTX1080Ti. The Adam optimizer was used to train the anomaly detection model and the root-mean-squared error function was used as the loss function during training. We employed a learning rate schedule of exponential decay with fixed steps and set the initial learning rate to 2.5 × 10^−5^.

### 4.3. Baseline Methods and Indicators Evaluation

Baseline methods: These state-of-the-art multivariate time series anomaly detection models, including DAGMM [39], MSCRED [41], USAD [45], MTAD-GAT [32], OmniAnomaly [29], GDN [30] and MAD-GAN [43] were used as baseline models in this paper for comparison with GTAD.

Evaluation indicators*:* Anomaly detection is a binary classification problem. Precision, recall and the F1 score were used to evaluate the detection performance of the model GTAD and various benchmark methods, as shown in Equation (7). Anomalous observations usually occur in the form of contiguous anomaly segments. If at least one observation of an anomalous segment is correctly detected, all the other observations of the segment are also considered as correctly detected, even if they were not. This approach is known as the point adjust method [5], which was utilized by our model.

(7) $$\mathrm{Precision}=\frac{TP}{TP+FP},\;\mathrm{Recall}=\frac{TP}{TP+FN},\;F1\;\mathrm{scores}=2\cdot \frac{\mathrm{Precision}\cdot \mathrm{Recall}}{\mathrm{Precision}+\mathrm{Recall}}$$

with *TP* referring to true positives, *FP* referring to false positives and *FN* referring to false negatives. Precision indicates how many of the anomalous events predicted by the algorithm are actual anomalous events. Recall denotes the percentage of predicted abnormal behavior versus all abnormal behavior. The F1 score is a better measure of model performance since it considers precision and recall. We expect the highest F1 score.

### 4.4. Results

We conducted experiments on the three publicly available datasets. In detail, we used the hyperparameters of the baseline models as presented in their respective papers. Table 2 shows the precisions, recalls and F1 scores of GTAD and all baseline methods. 

The experimental results showed that our model outperformed the other models on all three datasets. On the SMAP and SMD datasets, the F1 score of GTAD outperformed all benchmark models, and on the MSL dataset, it ranked second, only slightly behind the GDN, but the GDN did not work well on the SMAP and SMD datasets. Overall, compared with the best results of the baseline models, GTAD improved the F1 scores by about 7.4% on SMAP and 3.4% on SMD, which was significant in terms of anomaly detection. DAGMM could achieve better detection performance in SMD, but it did not work well in MSL and SMAP because DAGMM only considers the correlation between different sequences and ignores the temporal dependence. GTAD achieved better detection of anomalies by considering the temporal dependence using TCN. It shows that in the field of multivariate time series anomaly detection, obtaining temporal dependence helps with performance improvement.

MSCRED, UASD and OmniAnomaly discriminate anomalies only through reconstruction-based methods, which will result in some mild anomalies not being detected. The GDN performs anomaly detection via prediction only, focusing on feature engineering for the next timestamp, but it is always known that there are time series that are not predictable. Different data have different attributes, ranges and feature dimensions, resulting in different performances for the same algorithm on different datasets. Among them, MSCRED, GDN and OmniAnomaly performed the best on MSL and average on SMD and SMAP, while USAD worked well on SMD but had moderate detection in SMAP. GTAD performs anomaly detection by using a blend of prediction and reconstruction methods, with excellent results.

The MTAD-GAT based on a graph neural network (GNN) models time series as graph structures, which takes correlations between time series into consideration. However, dividing the time series into small sliding windows restricts the model from acquiring more contextual information, while causing the model to be more sensitive to the data and less effective in detecting datasets with long-term anomalies, like SMD, reducing its robustness. The MAD-GAN performed well on the SMD dataset with a large number of collective anomalies by considering the relationship between features through the autoencoder and adversarial training, but it ignored temporal information, resulting in moderate performance on the MSL and SMAP datasets with many contextual anomalies. GTAD applies TCN and multi-head attention to obtain more contextual information, which can improve the capability of the algorithm.

### 4.5. Ablation Analysis

**Figure 6:**
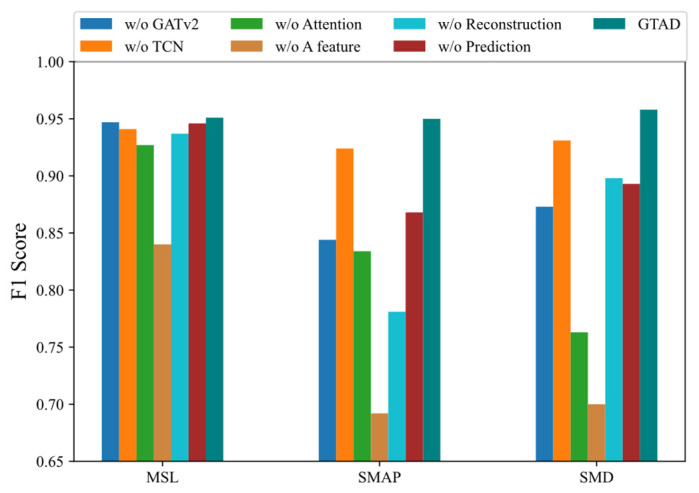
F1 score for GTAD and the model with one of the major components removed when used on the three datasets.

In this section, we present the results from analyzing the impact of six main components on the model performance: GATv2, TCN for feature extraction, attention mechanism, prediction and reconstruction errors in a feature, using the prediction method and employing the reconstruction method. On the three datasets, we observed the F1 score of the model after removing each principal component to measure its impact on the model. Specifically, the first variant of the model was a mapping of its own being used to replace GATv2. Second, an own mapping was chosen to replace the TCN in the feature extraction. Third, the multi-head attention layers were removed. Fourth, the error in predicting and reconstructing all features was chosen as the optimization objective and anomaly score, rather than choosing one of the dimensions. Fifth, we eliminated the reconstruction-based method for optimization and detection. Finally, we abandoned the prediction-based approach to optimization and detection. The following conclusions were obtained based on the results shown in Figure 6.

- Using the prediction and reconstruction errors of all dimensions as a loss function and anomaly detection resulted in an average decrease of about 23% in the F1 score. The most notable of these was a 26% decrease on the SMAP dataset, implying that the loss in selecting a dimension was significant.
- When we removed GATv2 from GTAD, the F1 scores decreased by about 6%, indicating that GTAD could work well using the GATv2, taking into account the correlation of the time series.
- Without the attention mechanism, the F1 scores were reduced by 10% on average. This suggested that adding the attention mechanism allowed for more contextual information and facilitated reconstruction.
- The absence of TCN caused a decrease of about 2% in the F1 score, indicating that the TCN could capture temporal dependence and local features that could steadily improve the model performance.
- Both the prediction-based and reconstruction-based methods were less effective on their own than the integration of the two methods, demonstrating that GTAD could combine their advantages.

### 4.6. Sensitivity Analysis of Hyperparameters

**Figure 7:**
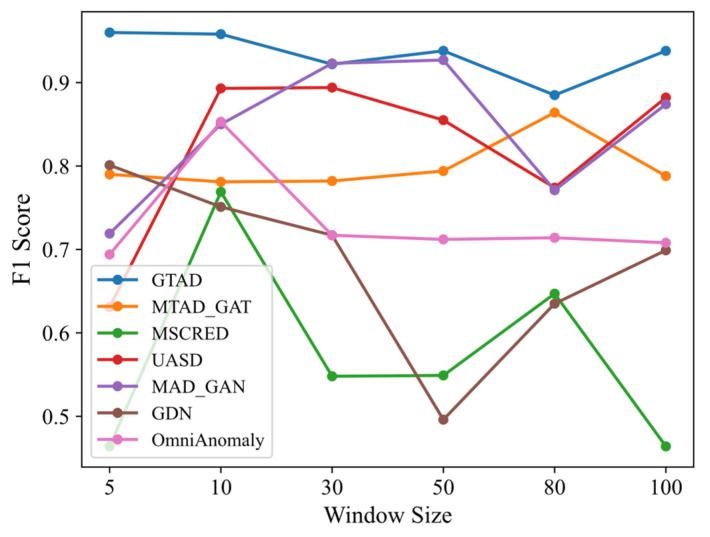
F1 score of GTAD and baseline models at different slide window sizes.

Sensitivity of the window size: We used the SMD dataset to compare the F1 scores of GTAD with its baseline approach under different sliding window sizes, as shown in Figure 7. Since DAGMM does not use sliding windows for data preprocessing, DAGMM was not addressed in this experiment. A small sliding window size will result in limited ability to obtain contextual information, but with a large sliding window size, short-term subtle anomalies will be hidden in long sequences, resulting in most baseline models being sensitive to sliding windows, e.g., GDN, MSCRED and USAD. Although MTAD-GAT and OmniAnomaly are also insensitive to sliding window size, their overall results were not as effective as GTAD. Long-term memory was preserved by the TCN, while contextual information was captured by a multi-headed attention mechanism, resulting in the low sensitivity of GTAD to the sliding window size.

**Figure 8:**
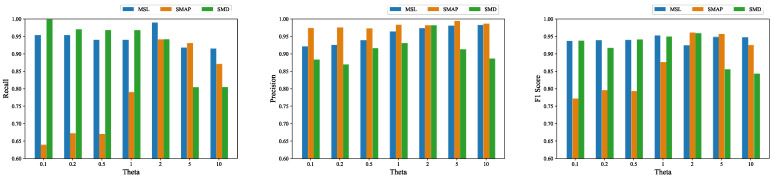
Recalls, precisions and F1 scores corresponding to different θ values on the three datasets.

Analysis of *θ*: Empirically, we adjusted the weights of the prediction and reconstruction errors of the training process without any significant improvement in the performance of our model; therefore, we made the prediction and reconstruction parts have the same weight in terms of the loss function. We performed an additional experiment to evaluate the recall, precision and F1 score of the algorithm for different values of *θ* on the three datasets, and the results are shown in Figure 8. The result showed that GTAD achieved the largest F1 scores on the SMAP and SMD datasets when *θ* was equal to two, and on the SML dataset when *θ* was equal to one. The recall was low when *θ* was less than one only on the SMAP dataset. Overall, GTAD achieved excellent anomaly detection performances at different values of *θ*, which indicated that our algorithm was robust against *θ*.

### 4.7. Overhead Analysis

In this section, we present the computational performance of GTAD by comparing it with all baseline models. Table 3 shows the average training time for all models on the three datasets in seconds per epoch. The training time for DAGMM and USAD, which consist of fully connected layers, was shorter than that of GTAD, and the time consumption of MSCRED was the largest. Models with LSTM or GRU, such as MSCRED, MTAD-GAT and OmniAnomaly, had a longer training time than GTAD with parallel computing. MSCRED first increases the amount of data by constructing a 2D feature matrix, and second, applies Conv-LSTM to process the data sequentially, leading to a tremendous time overhead. To ensure fairness in the experimental comparison, we set the batch size sequence length to be the same for all models except DAGMM. Because DGAMM does not consider the temporal relationship, its sequence length was set to 1, which also explained the short training time of DAGMM.

### 4.8. The Effectiveness of Automatic Threshold Selection

An efficient automatic thresholding method for time series anomaly detection is essential. In these three datasets, we compared the F1 scores obtained using the NDT method, which were obtained by iterating all thresholds in small steps between 0 and 2, with the best F1 score. The results shown in Table 4 indicate that the F1 scores of the NDT method were just lower than the best F1 scores (0.005 to 0.131), demonstrating the validity of the thresholding method used by GTAD.

### 4.9. Discussion

In this subsection, we discuss the advantages and disadvantages of our model. The advantages are mainly in three aspects. First, different from supervised learning for multivariate time series anomaly detection, GTAD does not require labels for each timestamp in the training of the model. Labels for time series are often generated manually by experts, which is often inefficient, time-consuming and costly. Moreover, unlike other joint reconstruction-based and prediction-based methods, we only reconstructed and predicted one sequence of multivariate time series without increasing the complexity of the multi-task optimization objective. This advantage was experimentally demonstrated in Section 4.5 to benefit anomaly detection. Finally, we implemented parallel operations using causal convolution and attention mechanisms in modeling time dependence and sequence correlation, respectively. Compared with models that process data sequentially, the experimental arguments in Section 4.6 and Section 4.7 revealed that our model had a short training time and low sensitivity to the time window size.

Although the proposed method showed very good performance, there are some limitations. A limitation of the proposed method is the inability of the model to explain the anomaly; we cannot provide the root cause of the anomaly occurrence. However, GDN, TranAD, MSCRED and OmniAnomaly enable the diagnosis and root cause analysis of the anomaly. Another limitation is that the training data of the model needs to be all normal data and cannot be mixed with abnormal data. However, DAGMM and many classical methods [17,19,23] do not require the training set to be all normal data.

## 5. Conclusions and Future Work

An effective anomaly detection algorithm can effectively reduce the cost of industrial production and the burden on operators. We proposed an unsupervised anomaly detection algorithm called GTAD for multivariate time series based on graph attention networks and temporal convolutional networks. By learning the temporal dependence of the series and correlations between different series, combining prediction and reconstruction optimization methods, and leveraging an automation threshold strategy, our model outperformed other state-of-the-art models on all three datasets. Future work will consist of two aspects. First, extending other GNN frameworks, such as gated graph neural networks and graph convolutional networks for time series anomaly detection, and second, providing a mechanism for anomaly diagnosis and analyzing the root cause of anomalies.

## Figures and Tables

**Table 1:** Description of the datasets. (%) is the percentage of anomalous data points in the dataset.

Dataset	Variables	Train	Test	Anomalies (%)
SMAP	25	135,183	427,617	13.13
MSL	55	58,317	73,729	10.27
SMD	38	708,405	708,420	4.16

**Table 2:** Experimental results on the MSL, SMAP and SMD datasets. The top 2 F1 scores are bolded.

Method	MSL		
	Precision	Recall	F1 Score
DAGMM	0.5412	0.9934	0.7007
MSCRED	0.8912	0.9862	0.9363
USAD	0.7949	0.9912	0.8822
MTAD-GAT	0.7917	0.9824	0.8767
OmniAnomaly	0.8867	0.9117	0.8989
GDN	0.9308	0.9892	**0.9591**
MAD-GAN	0.8517	0.8991	0.8747
GTAD	0.9668	0.9413	**0.9539**
**Method**	**SMAP**		
	**Precision**	**Recall**	**F1 Score**
DAGMM	0.5845	0.9058	0.7105
MSCRED	0.8175	0.9216	0.8664
USAD	0.7480	0.9627	0.8419
MTAD-GAT	0.7991	0.9991	**0.8880**
OmniAnomaly	0.7416	0.9776	0.8434
GDN	0.7480	0.9891	0.8518
MAD-GAN	0.8049	0.8214	0.8131
GTAD	0.9821	0.9426	**0.9620**
**Method**	**SMD**		
	**Precision**	**Recall**	**F1 Score**
DAGMM	0.9869	0.8174	0.8942
MSCRED	0.8164	0.7261	0.7686
USAD	0.9858	0.8174	0.8937
MTAD-GAT	0.7609	0.9999	0.8643
OmniAnomaly	0.8854	0.8827	0.8531
GDN	0.7754	0.7286	0.7513
MAD-GAN	0.9750	0.8827	**0.9265**
GTAD	0.9515	0.9690	**0.9601**

**Table 3:** Comparison of training times in seconds per epoch.

Methods	MSL	SMAP	SMD
DAGMM	3.06	7.04	37.36
MSCERD	231.47	416.51	3332.12
USAD	2.78	6.35	34.08
MTAD-GAT	3.91	8.72	43.89
OmniAnomaly	5.83	13.05	70.72
GDN	4.57	10.72	53.51
MAD-GAN	7.84	18.66	86.68
GTAD	3.73	7.16	37.96

**Table 4:** F1 scores obtained by the NDT vs. the best F1 scores.

Mothed	MSL	SMAP	SMD
F1 score-NDT	0.9539	0.9620	0.9601
F1 score-best	0.9544	0.9634	0.9732

## Data Availability

All the data used in the experiments can be downloaded from the following links: https://github.com/NetManAIOps/OmniAnomaly/tree/master/ServerMachineDataset/ (SMD dataset, accessed on 12 February 2021) and https://github.com/imperial-qore/TranAD/tr-ee/main-/data/SMAP_MSL (SMAP, MSL dataset, accessed on 3 March 2021).
